# RNA editing events and expression profiles of mitochondrial protein-coding genes in the endemic and endangered medicinal plant, *Corydalis saxicola*

**DOI:** 10.3389/fpls.2024.1332460

**Published:** 2024-02-06

**Authors:** Cui Li, Han Liu, Mei Qin, Yao-jing Tan, Xia-lian Ou, Xiao-ying Chen, Ying Wei, Zhan-jiang Zhang, Ming Lei

**Affiliations:** ^1^ National Center for Traditional Chinese Medicine (TCM) Inheritance and Innovation, Guangxi Botanical Garden of Medicinal Plants, Nanning, China; ^2^ Guangxi Key Laboratory of Medicinal Resources Protection and Genetic Improvement, Guangxi Botanical Garden of Medicinal Plants, Nanning, China; ^3^ Guangxi Engineering Research Center of Traditional Chinese Medicine (TCM) Resource Intelligent Creation, Guangxi Botanical Garden of Medicinal Plants, Nanning, China; ^4^ School of Basic Medical Sciences, Guangxi Medical University, Nanning, China; ^5^ Guangxi Key Laboratory for High-Quality Formation and Utilization of Dao-di Herbs, Guangxi Botanical Garden of Medicinal Plants, Nanning, China

**Keywords:** *Corydalis saxicola*, mitochondrial genome, RNA editing, mitochondrial plastid DNAs, expression profile

## Abstract

*Corydalis saxicola*, an endangered medicinal plant endemic to karst habitats, is widely used in Traditional Chinese Medicine to treat hepatitis, abdominal pain, bleeding hemorrhoids and other conditions. However, to date, the mitochondrial (mt) genome of *C. saxicola* has not been reported, which limits our understanding of the genetic and biological mechanisms of *C. saxicola*. Here, the mt genome of *C. saxicola* was assembled by combining the Nanopore and Illumina reads. The mt genome of *C. saxicola* is represented by a circular chromosome which is 587,939 bp in length, with an overall GC content of 46.50%. 40 unique protein-coding genes (PCGs), 22 tRNA genes and three rRNA genes were identified. Codon usage of the PCGs was investigated and 167 simple sequence repeats were identified. Twelve homologous fragments were identified between the mt and ct genomes of *C. saxicola*, accounting for 1.04% of the entire mt genome. Phylogenetic examination of the mt genomes of *C. saxicola* and 30 other taxa provided an understanding of their evolutionary relationships. We also predicted 779 RNA editing sites in 40 C*. saxicola* mt PCGs and successfully validated 506 (65%) of these using PCR amplification and Sanger sequencing. In addition, we transcriptionally profiled 24 core mt PCGs in *C. saxicola* roots treated with different concentrations of CaCl_2_, as well as in other organs. These investigations will be useful for effective utilization and molecular breeding, and will also provide a reference for further studies of the genus *Corydalis*.

## 1 Introduction

*Corydalis* DC., which contains approximately 530 species divided into 42 sections, is the largest genus within the Papaveraceae family (Zhang et al., 2008; Xu et al., 2022a; Chen et al., 2023b). *Corydalis* species are found across northern temperate regions, as well as the subarctic, subtropical Indochina and in the East Africa montane forests. Notably, the *Corydalis* species are particularly abundant around the Qinghai–Tibet Plateau and the Hengduan Mountains of China (Wu et al., 1999; Zhang et al., 2008). *Corydalis* species are adapted to wildly varied habitats, including riversides, forests, shrublands, grasslands, screes, cliffsides and high elevations (up to 6000 m). Such morphological diversity has made studying the taxonomy, ecology, evolutionary history and utilization of the genus challenging (Ohara and Higashi, 1994; Ohkawara et al., 1997; Kudo et al., 2001; Ehlers and Olesen, 2004; Niu et al., 2014; Zhang et al., 2014; Niu et al., 2017; Zhu et al., 2018; Chen et al., 2023b). Many *Corydalis* species possess brilliantly-colored flowers and are prized by horticulturalists and hobby gardeners (Lidén and Zetterlund, 1997). In addition, several *Corydalis* species are used as medicines to treat hepatitis, cancer, cardiovascular diseases and pain. These include *C. yanhusuo* W.T.Wang ex Z.Y.Su & C.Y.Wu, *C. decumbens* (Thunb.) Pers, *C. saxicola* Bunting, *C. ophiocarpa*, C. cava (L.) Schweigg. & Koerte and *C. tomentella* Franch, among others (Wu et al., 1999; Orhan et al., 2007; Kim et al., 2010; Novak et al., 2012; Li et al., 2013; Kim et al., 2014; Xu et al., 2022b; Feng et al., 2023; Qin et al., 2023b).

Among the medicinal *Corydalis* species, *C. saxicola* is widely used in Traditional Chinese Medicine due to its antibacterial, antiviral and anticancer activities (Orhan et al., 2007; Kim et al., 2010; Li et al., 2013; Kim et al., 2014; Tian et al., 2016; Zhang et al., 2016; Liu et al., 2019; Feng et al., 2023; Qin et al., 2023b). Studies suggest that these pharmacological activities are due to the presence of unique benzylisoquinoline alkaloid compounds known as cavidines (e.g., cavidine, apocavidine, dehydrocavidine and dehydroapocavidine) (Bhakuni and Chaturvedi, 1983; Qin et al., 2023a). As a calcicole plant, *C. saxicola* is found exclusively in and around the rocky crevices of calcium-rich karst landscapes. Unfortunately, increasing demand and a limited range have driven *C. saxicola* to become endangered. In order to safeguard *C. saxicola* as a medicinal resource, studies should be conducted to comprehensively evaluate the molecular biology and genetics of this endangered plant species.

Both mitochondria (mt) and chloroplasts (cp) have originated as prokaryotic endosymbionts, and these possess semi-autonomous genetic systems (Jiang et al., 2023a). The crucial physiological processes regulated by chloroplasts and mitochondria are photosynthesis and respiration, respectively (Raven, 2015). In the majority of seed plants, the mt and cp genomes are maternally inherited and are critical to growth and development (Pogson et al., 2015; Liberatore et al., 2016; Wang et al., 2022). In contrast to animal mt genomes, plant mt genomes have undergone numerous changes in the architecture, leading to a broad distribution in genome size, ranging from ~66 kb to ~12 Mb (Liu et al., 2014; Oldenburg and Bendich, 2015; Cole et al., 2018; Putintseva et al., 2020; Wu et al., 2022). In addition, plant mt genomes exhibit marked interspecies differences in gene order, gene length, gene content and migration of sequences from other organelles (Liu et al., 2014; Oldenburg and Bendich, 2015; Cole et al., 2018; Wu et al., 2022; Wang et al., 2024). Typically, mt genomes are circular, double-linked molecules. However, some plant mt genomes can exist as linear, multi-branched and polycyclic structures (Sloan, 2013; Gualberto et al., 2014; Kozik et al., 2019; Jackman et al., 2020). In comparison to cp genomes, these characteristics have made the assembly and exploration of plant mt genomes relatively difficult (Shan et al., 2023). To date (as of April 5, 2023), the National Center for Biotechnology Information (NCBI) database contains 602 published mt genomes, representing only approximately 5.7% of all cp genomes (10,479) (https://www.ncbi.nlm.nih.gov/genome/browse/#!/organelles/). Recently, the complete cp genomes of several *Corydalis* species, including *C. saxicola*, have been sequenced and published (Ren et al., 2021; Xu and Wang, 2021; Xu et al., 2022a). However, to date, only one *Corydalis* mt genome has been published (*C. pauciovulata*, Genbank: OR100522.1). Fortunately, the development of long-read PacBio and Nanopore sequencing techniques have made the sequencing of organellar genomes considerably simpler and faster.

In this study, the mt genome of *C. saxicola* was sequenced and annotated based on data obtained from Oxford Nanopore and Illumina. The relative synonymous codon usage (RSCU), sequence repeats and mitochondrial plastid DNAs (MTPTs) were analyzed. A phylogenetic analysis was performed between *C. saxicola* and 30 other angiosperm species based on 23 conserved mt protein-coding genes (PCGs). In addition, by combining PCR amplification and Sanger sequencing, we conducted an in-depth analysis of the RNA editing sites within the mt PCGs. Finally, we investigated the relative expression of 24 core mt PCGs in roots and other organs of *C. saxicola.* In addition, PCG expression was measured after treating the roots with exogenous CaCl_2_. These data will be invaluable to further research on the taxonomic classification, molecular evolution and breeding of *C. saxicola*.

## 2 Materials and methods

### 2.1 Plant materials, DNA and RNA extraction and sequencing

*C. saxicola* was identified by Drs Zhan-jiang Zhang and Cui Li of the Guangxi Botanical Garden of Medicinal Plants (GBGMP) (Nanning, Guangxi Zhuang Autonomous Region, China). Voucher specimens (reference number: 450123130505040LY) were deposited in the GBGMP Herbarium.

Fresh and healthy *C. saxicola* tissues were collected from the greenhouse located in the GBGMP experimental area. One gram of *C. saxicola* leaves were used four total DNA extraction according to the 2 × cetyltrimethylammonium bromide method (Porebski et al., 1997). The same qualified DNA sample was used for Illumina sequencing, Oxford Nanopore sequencing, and PCR amplification.

Thirty mg of *C. saxicola* roots, stems, branches, mature leaves and young leaves were used for total RNA extraction according to the manual of RNA Isolater Total RNA Extraction Reagent (Vazyme, China). The first-strand cDNA was synthesized using a HiScript III 1st Strand cDNA Synthesis Kit (+gDNA wiper) (Vazyme, China). Qualified extracted RNA was used as a template, and random hexamers and oligo(dT)_20_VN were used as primers. PCR was performed using 2 × Phanta Max Master Mix (Dye Plus) (Vazyme, China) and conducted on a T100 Thermal Cycler (BIO-RAD, USA).

### 2.2 Assembly and annotation of mt genomes

Flye (v.2.9.1-b1780) (Kolmogorov et al., 2019) was used to assemble the long reads of *C. saxicola*, with default parameters. The assembled contigs were then used to build a library using makeblastdb. BLASTn (Chen et al., 2015) was utilized to identify mt genome fragments, with the conserved mitochondrial genes of *Arabidopsis thaliana* were used as query sequences. With the help of BWA (v0.7.17) (Li and Durbin, 2009), the short reads and long reads were mapped to the contigs and all the mapped reads were retained. Finally, Unicycler (v0.5.0) (Wick et al., 2017) was employed to combine all mapped short and long reads for hybrid assembly, with default parameters. Unicycler-generated GFA format files were visualized with the help of Bandage (Wick et al., 2015).

Referring to previously published literature (Richardson et al., 2013), we found that the mt genome of *Liriodendron tulipifera* had the most complete mt gene content among angiosperms. As a result, in this study, the mt genome was annotated using GeSeq (v2.03) (Michael et al., 2017), with *L. tulipifera* (NC_021152.1) as the reference. IPMGA (http://www.1kmpg.cn/ipmga/) was also employed for annotation and integration of the results from both tools. Forty PCGs resulted from our annotation. For genes that were present in the *L. tulipifera* mt genome were not annotated in our newly sequenced species, and we further queried these using the BLASTn (v.2.13.0) (Chen et al., 2015) with parameters ‘-evalue 1e-10 -outfmt 6 -num_threads 10 -max_hsps 10’. tRNAscan-SE (v.2.0.11) (Lowe and Eddy, 1997) was also utilized to annotate the tRNA genes. Apollo (v1.11.8) (Lewis et al., 2002) was employed to manually adjust and rectify any annotation mistakes found in each mt genome.

### 2.3 Analysis of codon usage and repeated sequences

Protein-encoding sequences were extracted with PhyloSuite (v1.1.16) (Zhang et al., 2020). Codon preference analysis and RSCU value calculation were performed with Mega (v7.0) (Kumar et al., 2016). MISA (v2.1) (https://webblast.ipk-gatersleben.de/misa/; accessed on 5 August, 2023) (Beier et al., 2017), TRF (v4.09) (https://tandem.bu.edu/trf/trf.unix.help.html; accessed on 6 August, 2023) (Benson, 1999) and REPuter (https://bibiserv.cebitec.uni-bielefeld.de/reputer/; accessed on 8 August, 2023) (Kurtz et al., 2001) were employed to detect the repetitive sequences. These included microsatellite sequence repeats, tandem repeats (TR) and dispersed repeats. The results were visualized using Excel (2021) and the Circos package (v0.69-9) (Zhang et al., 2013).

### 2.4 Identification of MTPTs, and RNA editing events

The cp genome of *C. saxicola* was assembled using GetOrganelle (v1.7.7.0) (Jin et al., 2020) and annotated using CPGAVAS2 (Shi et al., 2019). CPGView (Liu et al., 2023) was employed to rectify annotation mistakes in the cp genomes. BLASTn (v.2.13.0) (Chen et al., 2015) was utilized to identify homologous fragments in the mt and cp genomes, and the results were visualized using the Circos package (v0.69-9) (Zhang et al., 2013). Putative RNA editing sites were predicted using Deepred-mt (Edera et al., 2021), with a cutoff value of 0.9.

### 2.5 Phylogenetic and synteny analyses

The mt genome sequences of closely related plant species were obtained from NCBI (https://www.ncbi.nlm.nih.gov; accessed on 4 August, 2023), and PhyloSuite (v1.1.16) (Zhang et al., 2020) was then employed to extract common genes. To conduct a phylogenetic analysis, the nucleotide sequences of shared genes were aligned using MAFFT (v7.505) (Katoh and Standley, 2013), and the aligned sequences were concatenated to form a data matrix. Based on this data matrix, the maximum likelihood analysis was performed to construct the phylogenetic tree using IQ-TREE (v1.6.12) (Nguyen et al., 2015), with a bootstrap value of 1000, and then visualized using ITOL (v6) (Letunic and Bork, 2019).

To investigate the collinearity of the mt genomes, BLASTn (Chen et al., 2015) was used to identify conserved homologous sequences between *C. saxicola* and other closely related plant species, with the following parameters: -evalue 1e-5, -word_size 9, -gapopen 5, -gapextend 2, -reward 2, and –penalty 3. Only collinear blocks of at least 500 bp in length were selected for further analysis. MCscanX (Wang Y. et al., 2012) was then used to generate a multiple synteny plot.

### 2.6 RT-qPCR and heatmap generation

The first-strand reaction products were diluted with sterilized distilled H_2_O three times, and the diluted products (1 µL) were used for qPCR (20 µL). qPCR was performed using ChamQ Universal SYBR qPCR Master Mix (Vazyme, China) and conducted on a QuantStudio 3 system (Thermo Fisher Scientific, USA). Three biological replicates and three technical replicates were performed. The relative expression levels of specific genes were calculated using the 2^−ΔΔCt^ method, with *glyceraldehyde-3-phosphate dehydrogenase 8* (*CsGAPDH8*) used as the internal control (Livak and Schmittgen, 2001). All primers used for qPCR are listed in **Supplementary Table S1**. The relative expression levels were visualized using tbtools (v2.008) (Chen et al., 2023a).

### 2.7 CaCl_2_ treatment of *C. saxicola*

To test CaCl_2_ response, one-month-old of *C. saxicola* plants grown in pots containing a matrix with a 3:1 mixture ratio of peat soil and vermiculite in the greenhouse (23 °C, 16 h light, a photo flux density of 120 µmol m^-2^ s^-1^) were treated with equal volume of CaCl_2_ at 4 mmol/L, 30 mmol/L, 100 mmol/L, 200 mmol/L, 300 mmol/L and 400 mmol/L for 25 d. The same batch of plants treated with the same quantity of water were set as the control (CK). The organs were then cleaned and physically isolated and immediately frozen or dried for further research.

### 2.8 Measurement of dehydrocaverdine content

Agilent 1260 Infinity II (Agilent Co. Ltd, NY, USA) was used to determine the relative content of dehydrocaverdine. 200.0 mg of dried plant tissue was weighed in a 15-mL tube and 10 mL of methanol was added. The samples were ultra-sonicated for 60 min, and then centrifuged at 13,000 g for 15 min. The supernatant was transferred to a new 15-mL tube and dried using nitrogen pressure reduction method at room temperature. The dried sample was then dissolved with 1-mL of mobile phase (acetonitrile: 0.01% of K_2_HPO4 aqueous solution = 21:79 (v/v)) and passed through a 0.22 μm filter for measurement. Dehydrocaverdine was eluted under the following conditions: Agilent XDB C_18_ chromatographic column (150 mm × 4.6 mm, 5 µm) (Agilent Co. Ltd, NY, USA); mobile phase (acetonitrile: 0.01% of K_2_HPO4 = 21:79 (v/v)); 30 °C; 347 nm of detection wavelength; 1.0 mL/min of volume flow rate. A standard sample of dehydrocaverdine was purchased from Shanghai Yuanye Co. Ltd (Shanghai, China).

## 3 Results

### 3.1 General features of *C. saxicola* mt genome

**Figure 1:**
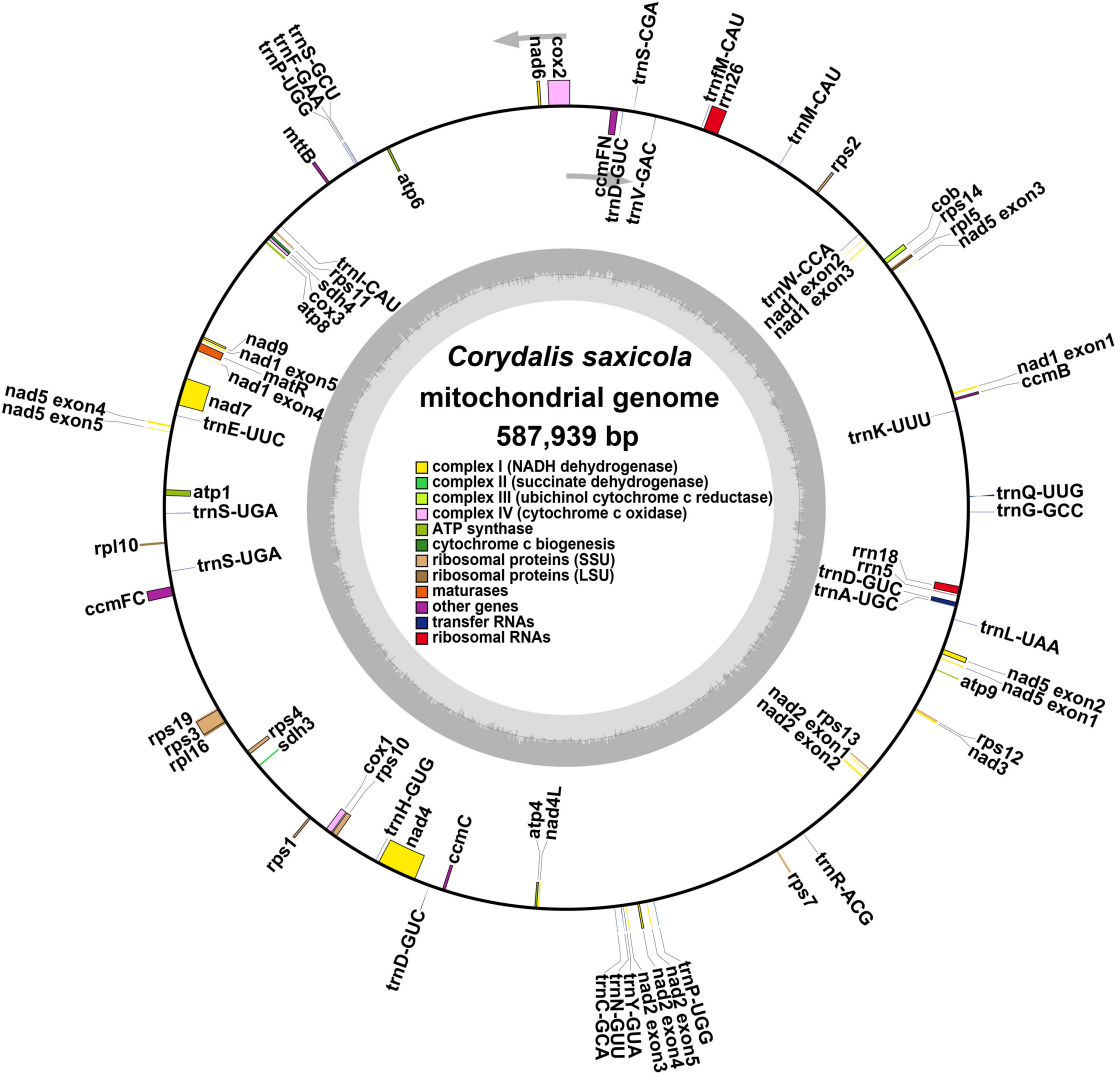
A map of the *C. saxicola* mt genome. The genes drawn inside and outside the circle are transcribed clockwise and counterclockwise, respectively.

The *C. saxicola* mt genome was assembled using a hybrid assembly of both long and short reads. Finally, a graphical mt genome comprising 3 nodes (contig1, contig2 and contig3) was constructed by resolving the repeat regions (**Supplementary Figure S1**). Contig3 was predicted to be repeats, as its depth was approximately twice when compared with contig1 and contig2 (**Supplementary Figure S1**; **Supplementary Table S2**). Finally, we derived a single circular molecule for the *C. saxicola* mt genome (Genbank: OR625126) (**Figure 1**).

The *C. saxicola* mt genome is 587,939 bp in length, with an overall GC content of 46.50%. A total of 40 unique PCGs were identified, including 24 core genes and 16 variable genes (**Table 1**). In addition, 22 tRNA genes and three rRNA genes were annotated. All the PCGs and rRNA genes, and most of the tRNA genes, occurred as single copies. However, two tRNA genes (*trnP-UGG* and *trnS-UGA*) occurred as double copies, and one tRNA gene (*trnD-GUC*) occurred as a triple copy (**Table 1**). Among the 65 annotated genes, 9 genes contained more than one exon. Of these, 3 (*ccmFC*, *rps3* and *rps10*), 1 (*cox2*), 1 (*nad4*) and 4 (*nad1*, *nad2*, *nad5* and *nad7*) contained 2, 3, 4 and 5 exons, respectively (**Supplementary Table S3**).

**Table 1:** A list of genes identified in the *C. saxicola* mt genome.

	Gene group	Gene name
Core genes	ATP synthase	*atp1, atp4, atp6, atp8, atp9*
	NADH dehydrogenase	*nad1, nad2, nad3, nad4, nad4L, nad5, nad6, nad7, nad9*
	Cytochrome *b*	*cob*
	Cytochrome *c* biogenesis	*ccmB, ccmC, ccmFC, ccmFN*
	Cytochrome *c* oxidase	*cox1, cox2, cox3*
	Maturases	*matR*
	Protein transport subunit	*mttB*
Variable genes	Ribosomal protein large subunit	*rpl5, rpl10, rpl16*
	Ribosomal protein small subunit	*rps1, rps2, rps3, rps4, rps7, rps10, rps11, rps12, rps13, rps14, rps19*
	Succinate dehydrogenase	*sdh3, sdh4*
rRNA genes	Ribosome RNA	*rrn5, rrn18, rrn26*
tRNA genes	Transfer RNA	*trnA-UGC, trnC-GCA, trnD-GUC* (×3), *trnE-UUC, trnF-GAA, trnfM-CAU, trnG-GCC, trnH-GUG, trnI-CAU, trnK-UUU, trnL-UAA, trnM-CAU, trnN-GUU, trnP-UGG* (×2), *trnQ-UUG, trnR-ACG, trnS-CGA, trnS-GCU, trnS-UGA* (×2), *trnV-GAC, trnW-CCA, trnY-GUA*

(×2) and (×3) refer to genes with two and three copies, respectively.

In addition, we also produced the complete *C. saxicola* cp genome (Genbank: OR625127). The cp genome was 190,564 bp in length and contained a pair of inverted repeat (IR) regions (47,692 bp each), a large single-copy (LSC) region (96,266 bp), and a small single-copy (SSC) region (8,890 bp) (**Supplementary Figure S2**). A total of 110 genes were identified, including 77 PCGs, 29 rRNA genes and 4 tRNA genes (**Supplementary Table S4**). This cp genome was subsequently used for comparative analysis with the *C. saxicola* mt genome in order to identify the MTPTs.

### 3.2 Codon usage of PCGs

**Figure 2:**
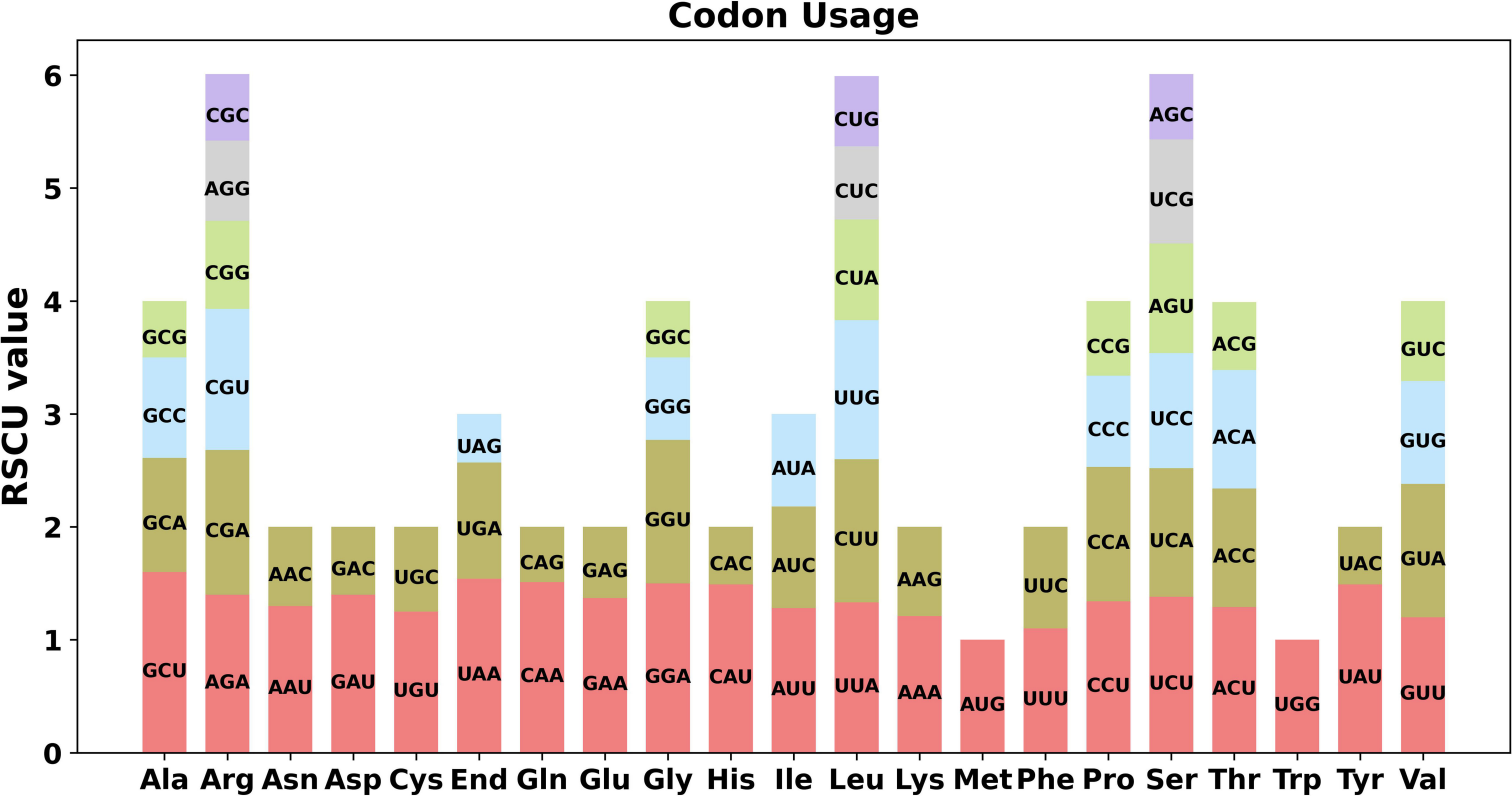
Codon usage of 20 amino acids and stop codons across all *C. saxicola* mt PCGs. The codons are represented by different colors in the histogram.

The 64 codons present in eukaryotic genomes are capable of encoding 20 different amino acids, among which Met and Trp are the only two that are not encoded by multiple codons. To investigate codon usage preference, we performed codon usage analysis on the 40 PCGs identified in the *C. saxicola* mt genome (**Figure 2**, **Supplementary Table S5**). With the exception of the start codon (AUG) and tryptophan (Trp, UGG), both of which had a RSCU of one, we observed a shared preference for codon usage in *C. saxicola* mt PCGs. Specifically, alanine (Ala) was preferentially encoded by GCU, with a RSCU of 1.60. The stop codon was preferentially encoded by UAA, with a RSCU of 1.54. Interestingly, phenylalanine (Phe) had a RSCU of 1.2, showing no clear codon usage preference.

### 3.3 Repeated sequences

We identified 167 SSRs across the *C. saxicola* mt genome, including 41 mononucleotide, 42 dinucleotide, 17 trinucleotide, 61 tetranucleotide, five pentanucleotide and one hexanucleotide SSRs (**Supplementary Figure S3A**; **Supplementary Table S6**). Together, monomeric and dimeric SSRs made up 49.70% of all SSRs (**Supplementary Figure S3A**). Of the 13 monomeric SSRs, 53.85% (7) were adenine (A) monomeric repeats, and of the dimeric SSRs, 29.03% were TR (**Supplementary Figure S3A**). Tetrameric SSRs were the most common, representing 36.53% (61) of all SSRs (**Supplementary Figure S3A**; **Supplementary Table S6**). In addition, 17 TR, ranging from 12 to 42 bp in length, with a matching degree greater than 69%, were identified in the *C. saxicola* mt genome (**Supplementary Figure S3B**; **Supplementary Table S7**). Moreover, 797 pairs of dispersed repeats of at least 30 bases in length were identified, including 388 pairs of palindromic repeats, 408 pairs of forward repeats and one pair of reverse repeats (**Supplementary Figure S3B**; **Supplementary Table S8**). No complementary repeats were detected. The longest pairs of palindromic and forward repeats were 1559 bp and 4268 bp, respectively (**Supplementary Table S8**).

### 3.4 Identification of MTPTs

**Figure 3:**
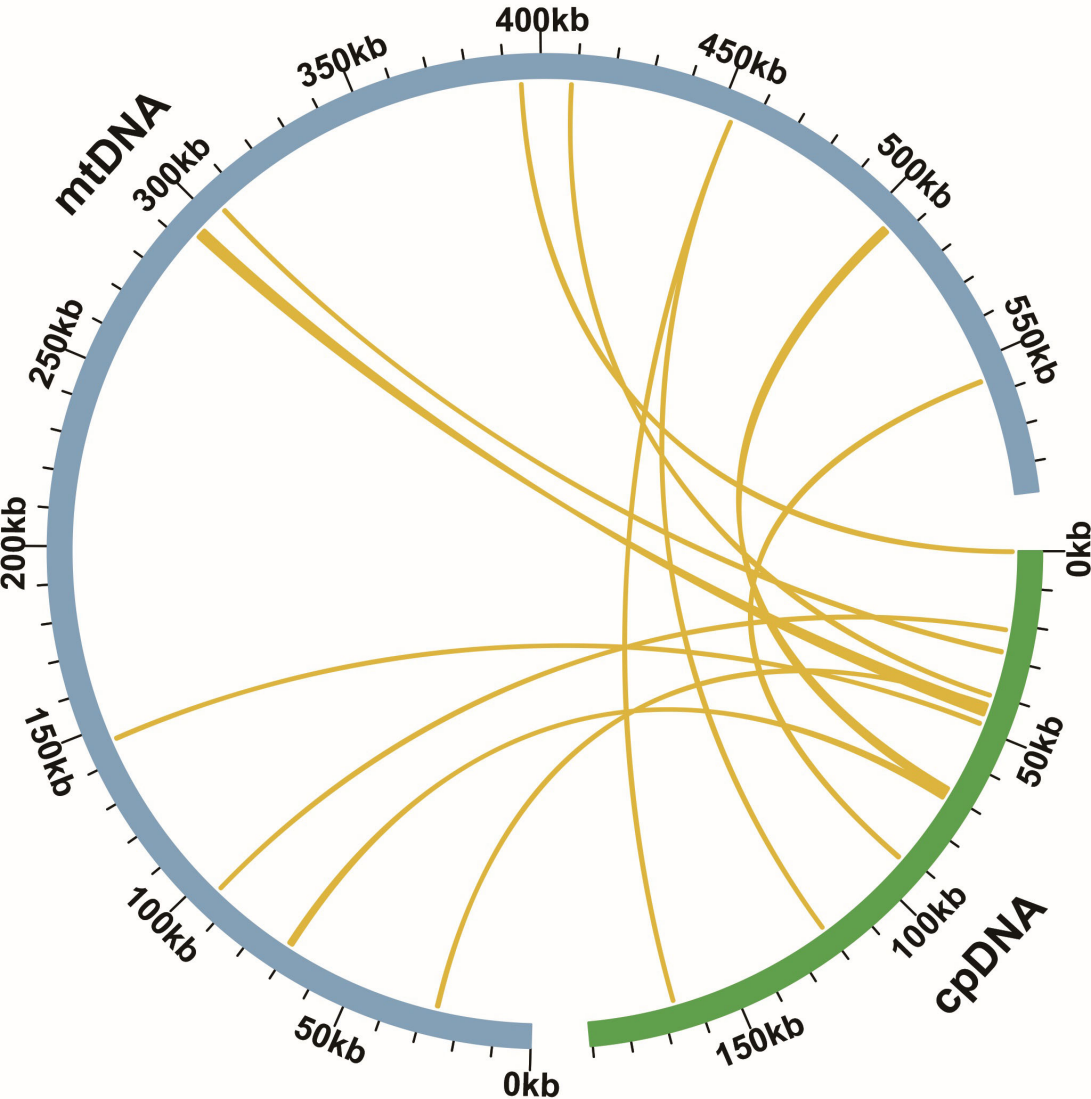
Homologous fragment analysis of *C. saxicola* cp and mt genomes. The blue and green arcs represent the mt genome and cp genome, respectively. The yellow lines correspond to homologous genomic fragments.

According to sequence similarity analysis, 12 homologous fragments were identified between the *C. saxicola* mt and ct genomes (**Figure 3**). The homologous fragments were 6135 bp in length, accounting for 1.04% of the entire mt genome (**Supplementary Table S9**). Among them, three fragments exceeded 1000 bp in length (**Supplementary Table S9**). These fragments were sourced from PCGs, tRNA genes and intergenic regions of the plastid genome (**Supplementary Table S9**). Specifically, 7 fragments were associated with either 13 partial or complete plastid PCGs, including *psaB* (partial), *psbC* (partial), *psbD* (partial), *psbJ* (partial), *rpl14* (partial), *rpoC2* (partial), *petG*, *petL*, *psbE*, *psbF*, *psbL* and *psbZ* (**Supplementary Table S8**). In addition, 7 homologous fragments encompassed 7 complete tRNA genes, including *trnD-GUC*, *trnH-GUG*, *trnN-GUU*, *trnM-CAU*, *trnP-UGG*, *trnS-UGA* and *trnW-CCA* (**Supplementary Table S9**).

**Figure 4:**
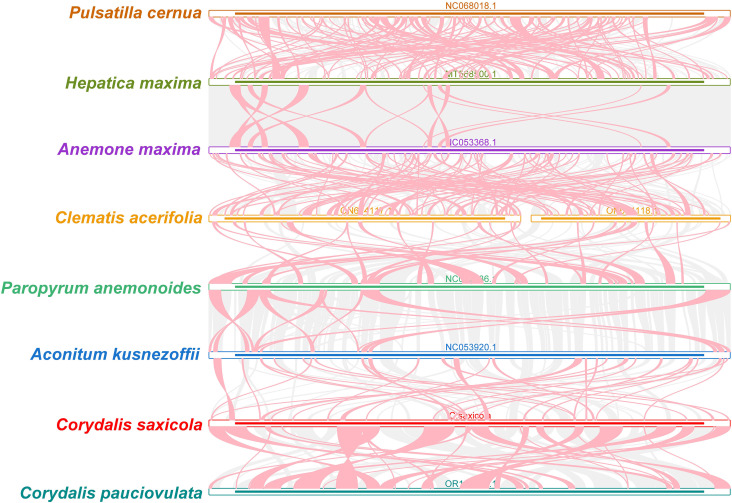
*C. saxicola* mt genome synteny. The bars signify the different mt genomes, with ribbons representing the homologous sequences between adjacent species. The red areas indicate where inversions have occurred, while the gray areas indicate regions of high homology. Blocks of less than 0.5 kb shared between species are not shown. Regions lacking a common block signify that they are exclusive to the species.

By analyzing sequence similarities, we created a multiple synteny plot of *C. saxicola* with 7 other related species. Several homologous, co-linear blocks were identified between *C. saxicola* and its relatives, particularly *C. pauciovulata* (**Figure 4**). Additionally, several regions that are exclusive to *C. saxicola* were discovered and these do not share similarities with other species. Moreover, we discovered that the co-linear blocks were not in the same order among the 8 species, indicating that extensive genomic rearrangements had occurred.

### 3.5 Phylogenetic and synteny analyses

**Figure 5:**
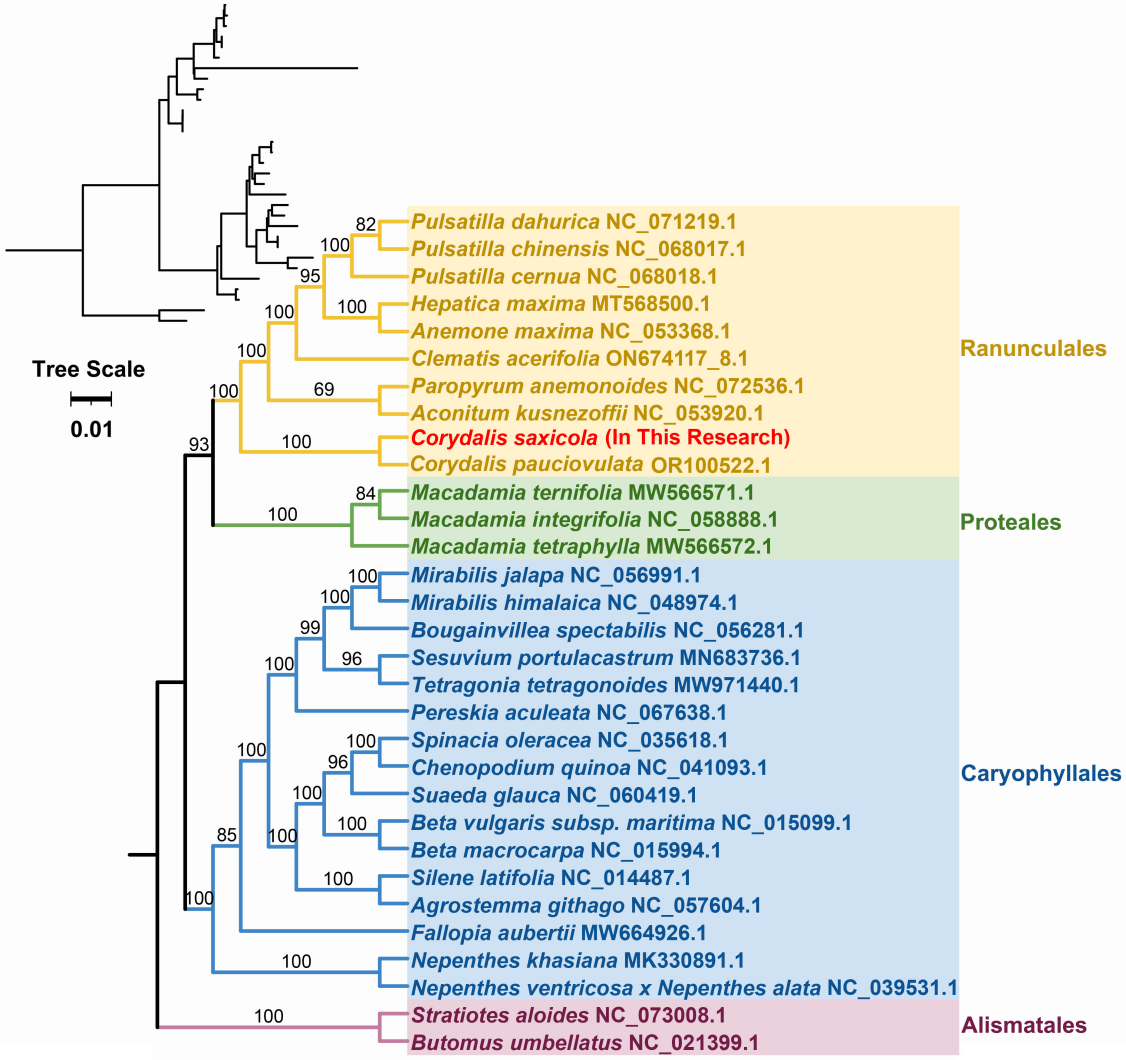
Phylogenetic analysis of *C*. *saxicola* and 30 other species based on the nucleotide sequences of 23 conserved core mt PCGs. *S. aloides* and *B. umbellatus* were selected as outgroups. The number at each node represents the bootstrap probability.

To explore the evolutionary history of *C. saxicola*, we constructed a phylogenetic tree using the nucleotide sequences of 23 conserved core mt PCGs (*atp1*, *atp4*, *atp6*, *atp8*, *atp9*, *ccmB*, *ccmC*, *ccmFC*, *ccmFN*, *cob*, *cox2*, *cox3*, *matR*, *nad1*, *nad2*, *nad3*, *nad4L*, *nad5*, *nad*6, *nad7*, *nad9*, *rps3* and *rps12*) from 31 species representing four orders (Ranunculales, Proteales, Caryophyllales and Alismatales) (**Figure 5**, **Supplementary Table S10**). The mt genomes of *Stratiotes aloides* and *Butomus umbellatus* were selected as outgroups. Overall, the phylogenetic topology obtained from mt DNA corresponded to the most recent Angiosperm Phylogenetic Group (APG) classification (**Figure 5**). Unsurprisingly, *C. saxicola* (Papaveraceae family, Ranunculales order) was most closely related to *C. pauciovoulata* (**Figure 5**).

### 3.6 RNA editing events in *C. saxicola*

**Figure 6:**
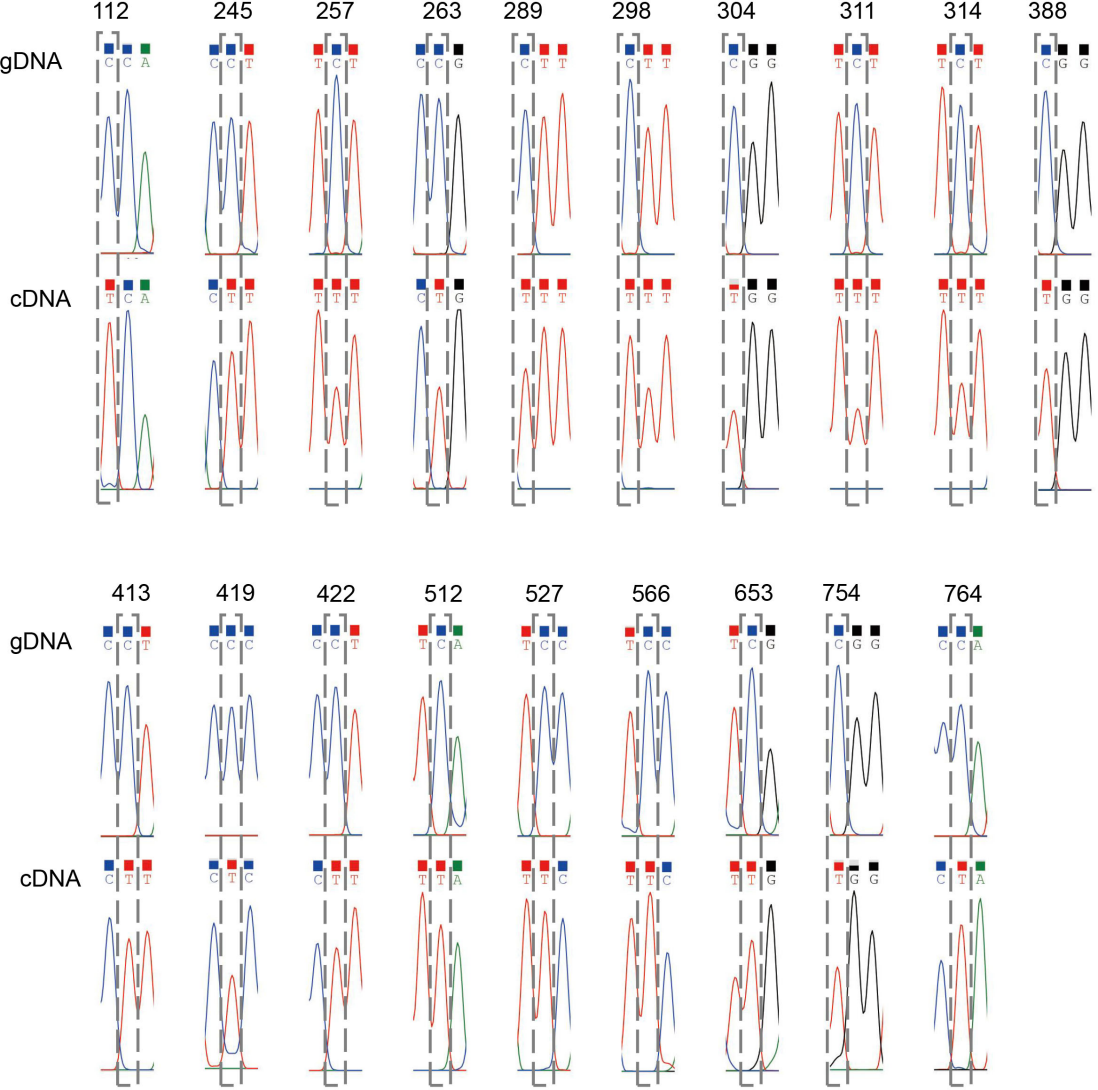
Validation of 19 RNA editing sites within *cox3*. Each chromatogram displays the sequences before and after editing at the RNA editing site. The position of each site is indicated at the top and framed by grey dashed rectangles.

To identify potential RNA editing sites amongst the 40 C*. saxicola* mt PCGs, we carried out PCR amplification and Sanger sequencing of the genomic DNA (gDNA) and complementary DNA (cDNA) of all 40 PCGs. Primers were designed for flanking sequences of at least 40 bp upstream from either the start codon or downstream from the stop codon. If the gDNA contained one or more exons, primers were designed for flanking sequences upstream and downstream of each exon. The PCR amplification results are shown in **Supplementary Figure S4**, and the detailed primer information is listed in **Supplementary Table S11**. In general, the positions which varied between the gDNA and cDNA amplification products from the same gene indicated the presence of RNA editing sites. In all, we identified 546 ‘C to U’ RNA editing sites (**Supplementary Tables S12**, **S13**, **Supplementary File A**). Validation for 19 RNA editing sites within the *cox3* gene is shown in **Figure 6**. Among the RNA editing sites, 25 (4.6%) were synonymous substitution sites and 516 were non-synonymous substitution sites (**Supplementary Table S12**). Two of RNA editing sites introduced termination codons into *atp9* and *rps10* (**Supplementary Table S12**). Among all the mt PCGs, *nad4* contained the greatest number of RNA editing sites (54), while *mttB* and *ccmB* contained 46 and 43 editing events, respectively (**Supplementary Tables S12**, **S13**). Interestingly, no RNA editing sites were identified in *rps11* and *rps12* (**Supplementary Tables S12**, **S13**). Eight types of synonymous substitutions were identified, including Asp to Asp (1), Gly to Gly (1), Ile to Ile (6), Leu to Leu (4), Phe to Phe (6), Pro to Pro (3), Ser to Ser (3) and Tyr to Tyr (1) (**Supplementary Table S12**). Additionally, 14 types of non-synonymous substitutions were identified, including Ala to Val (4), Arg to Cys (43), Arg to stop codon (2), Arg to Trp (32), His to Tyr(22), Leu to Phe (19), Phe to Leu (1), Pro to Leu (119), Pro to Phe (12), Pro to Ser (48), Ser to Leu (117), Ser to Phe (81), Thr to Ile (10) and Thr to Met (7) (**Supplementary Table S12**).

**Figure 7:**
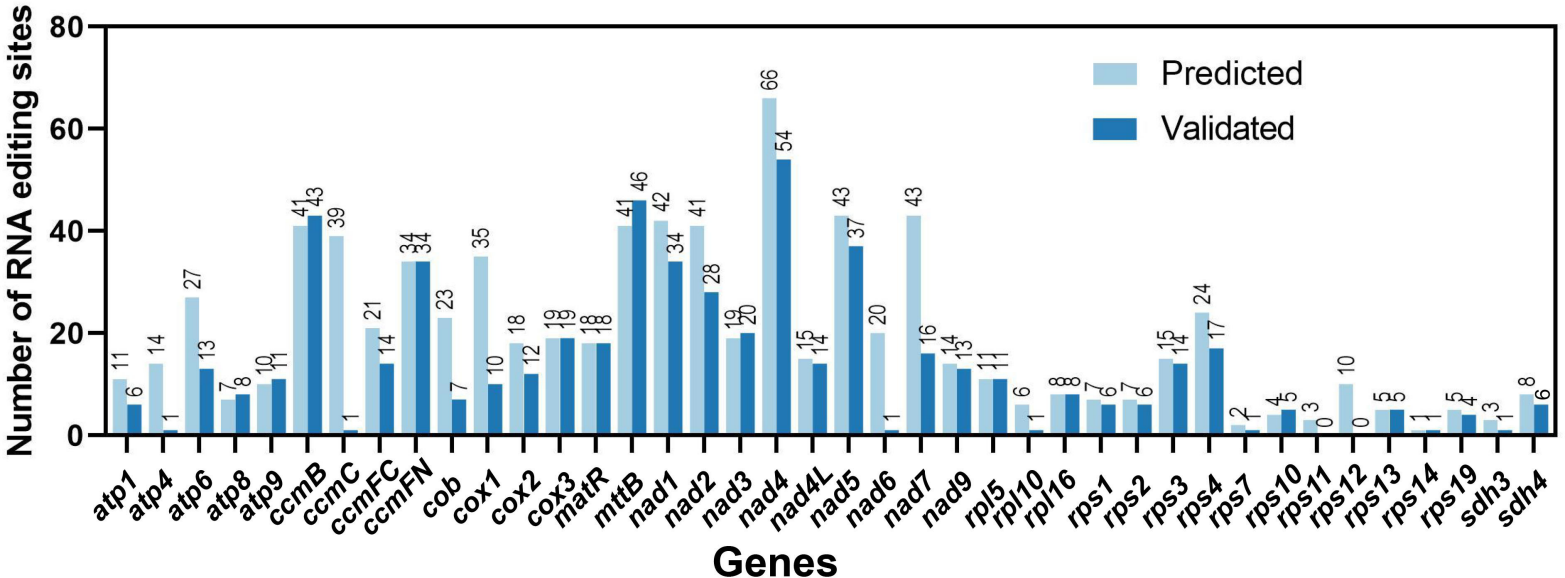
Prediction and validation of RNA editing sites among the 40 *C. saxicola* mt PCGs.

We further compared the validated RNA editing sites with those predicted by Deepred-mt (Edera et al., 2021), using a cutoff value of 0.9. A total of 779 RNA editing sites were predicted, and all were C-U (**Figure 7**; **Supplementary Table S12**). Among these predicted RNA editing sites, 506 (65.0%) were validated. Notably, 38 other RNA editing sites were validated but not predicted (**Supplementary Table S12**).

### 3.7 Transcript profiles of 24 core mt PCGs

**Figure 8:**
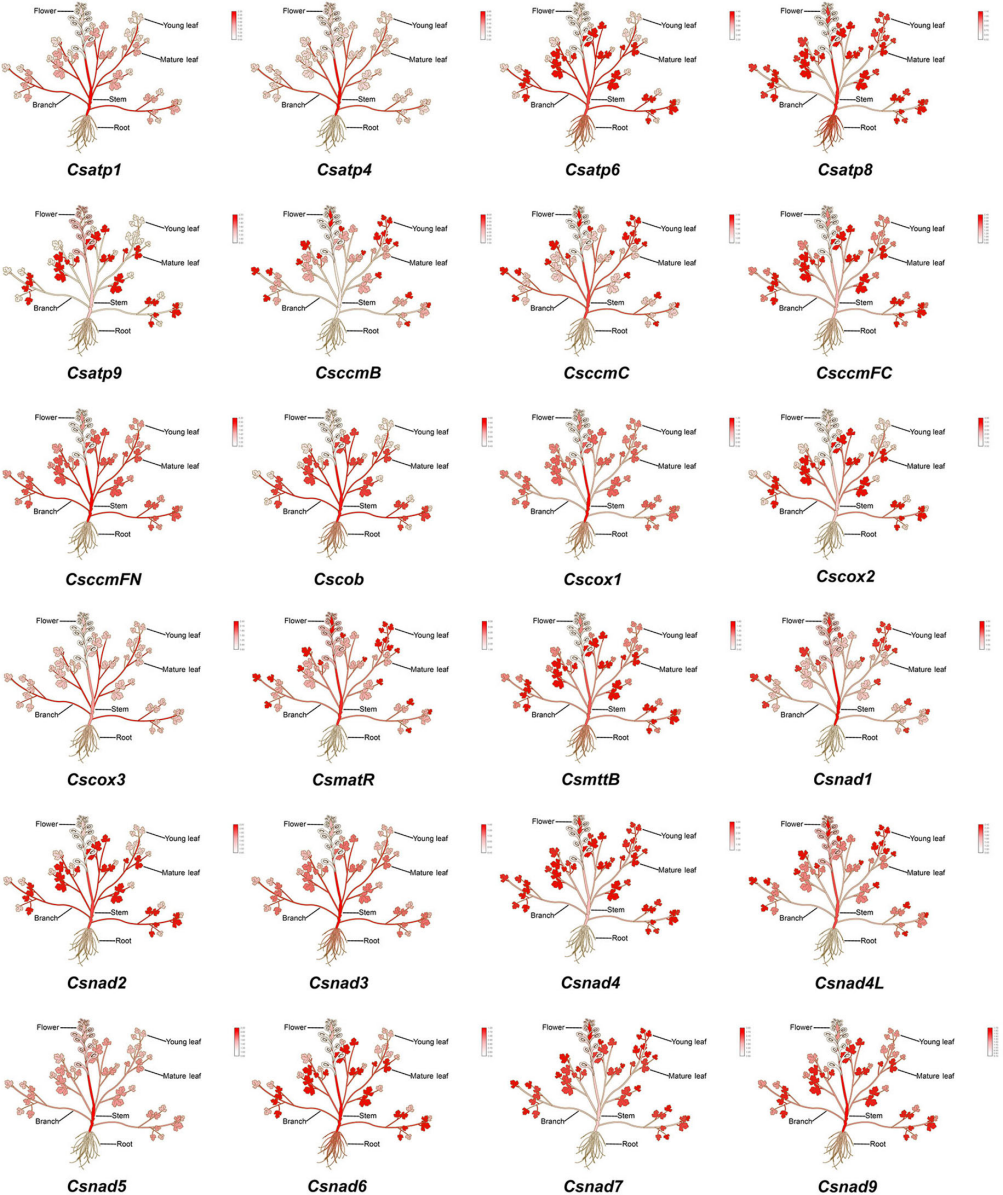
A heatmap of the relative expression levels of 24 core mt PCGs in the roots, stems, branches, mature leaves, young leaves and flowers of *C. saxicola*, as determined by RT-qPCR.

To functionally characterize 24 core *C. saxicola* mt PCGs, we studied their expression profiles in various tissues via reverse transcription followed by quantitative real-time PCR (RT-qPCR). While transcriptional expression levels differed between the genes, the majority were minimally expressed in roots and flowers (**Figure 8**).

**Figure 9:**
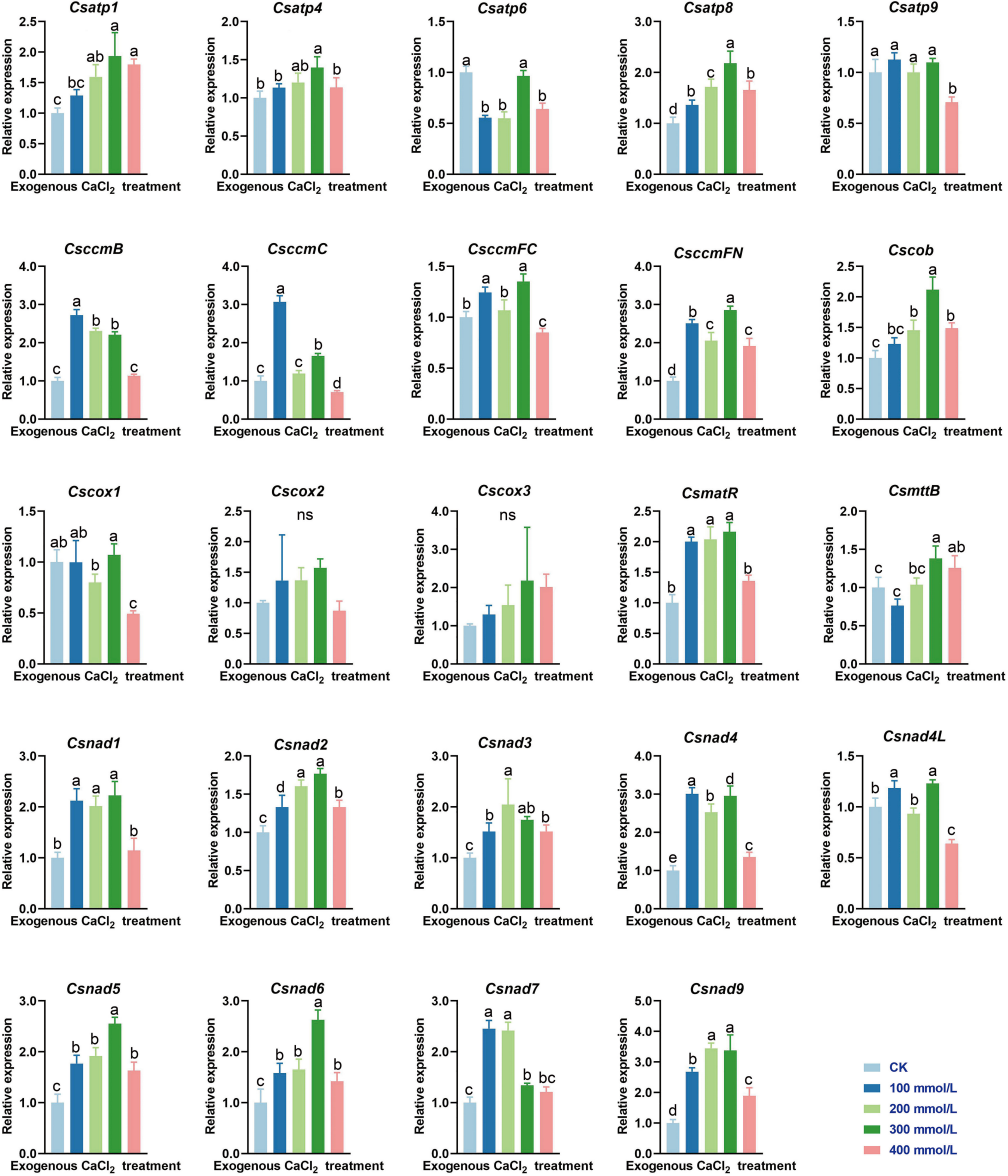
The relative expression levels of 24 core mt PCGs in *C. saxicola* roots grown under CaCl_2_ stress. The data are shown as the means ± SD of three replicates. Different lowercase letters indicate significant differences among treatments (ANOVA); differences were considered significant at *p* < 0.05.

Karst habitats are distinguished by their calcium-rich limestone background, and this environment is home to many calcicole plants (Xue et al., 2018). As a plant native to karst regions, *C. saxicola* is particularly resistant to high concentrations of calcium. To investigate whether any of the *C. saxicola* mt PCGs are responsive to exogenous CaCl_2_, the expression levels of 24 core mt PCG transcripts were analyzed in *C. saxicola* roots by RT-qPCR. Interestingly, exogenous calcium resulted in the marked upregulation of the majority of the tested genes, with the exception of *atp6*, *atp9*, *cox1*, *cox2* and *cox3* (**Figure 9**). Compared to that in CK, the expression levels of *atp6* were significantly lower in 100, 200, and 400 mmol/L CaCl_2_ treatments, while those of *atp9* and *cox1* were only significantly lower in 400 mmol/L CaCl_2_ treatments. However, the quantities of *cox2* or *cox3* transcripts in CaCl_2_ treatments were non-significantly different from CK.

## 4 Discussion

The study of mitochondria started in earnest around 1950 with the first isolation of mitochondria from animal and plant tissues (Møller et al., 2021). Mitochondria are essential for cellular functioning and are responsible for generating energy and mediating interactions between other organelles (Wang et al., 2022). Besides their role in respiration, plant mitochondria are actively engaged in a range of other cellular activities, such as photosynthesis as well as Crassulacean acid and C_4_ metabolism, heat generation, temperature regulation, immunity, biotic and abiotic stress resistance, programmed cell death and genomic evolution (Møller et al., 2021; Welchen et al., 2021; Le et al., 2022; Wang et al., 2022). As is the case with other eukaryotes, plant mitochondria are encased by two membranes and contain their own genetic material, the mt genome (Robles and Quesada, 2021). Similar to the cp genome, that of the mt exhibits a distinct organization and pattern of inheritance which is unlike that of the nuclear genome (Robles and Quesada, 2021). In addition, the mitochondria of animals and plants differ in several ways. First, many animal cells possess more than one mt genome copies per mitochondrion. However, plant cells contain mtDNA at a much lower rate than one copy per mitochondrion (Zhang et al., 2023). For example, direct observations via super-resolution microscopy suggest that human fibroblast cells contain at least 1.4 copies of the mt genome per mitochondrion (Kukata et al., 2011), while only one-third of *A. thaliana* leaf mitochondria contain mtDNA (Wang et al., 2010). Second, plant mt genomes range from ~66 kb to ~12 Mb, while animal mt genomes are far less variable in length (Wang et al., 2010). For example, mammalian mt genomes are generally 15-17 kb in length (Wang et al., 2010). Third, plant mitochondria often contain only partial mt genomes (Preuten et al., 2010; Wang et al., 2010; Rose, 2021). Finally, plant mt genomes exhibit diverse structures, including circular double-linked molecules as well as linear, multi-branched and polycyclic structures (Sloan, 2013; Gualberto et al., 2014; Kozik et al., 2019; Jackman et al., 2020). Such complexity has limited the comprehensive study of plant mt genomes.

### 4.1 Overview of the *C. saxicola* mt genome

Fortunately, the development of PacBio and Oxford Nanopore long-read sequencing techniques has made the sequencing of organellar genomes simpler and faster. However, assembling plant mt genomes often remains challenging due to their structural variety and numerous repetitive sequences. Here, we successfully assembled the mt genome of *C. saxicola* based on Oxford Nanopore and Illumina data. The *C. saxicola* mt genome is represented by a single-circle chromosome, 587,939 bp in length, and includes 65 genes (**Figure 1**; **Table 1**). These characteristics are similar to the closely related *C. pauciovulata*, which is the first mt genome to be published for any of the *Corydalis* species and it was also a single-circle structure, 675,483 bp in length and included 66 genes. To date, the largest plant mt genome, ~11.7 Mbp, has been recorded from Siberian larch (*Larix sibirica* Ledeb.) (Putintseva et al., 2020), which is approximately 21 times of the *C. saxicola* mt genome in size. Phylogenetic analysis indicated that *C. saxicola* is most closely related to *C. pauciovoulata* (**Figure 5**), and they share many homologous and co-linear blocks (**Figure 4**). These results indicate that our assembly and annotation of *C. saxicola* mt genome were reliable, and that genome structure, genome arrangement and gene number may be conserved to some extent among the *Corydalis* species, at least in *C. saxicola* and *C. pauciovoulata*. However, more mt genomes of *Corydalis* or Papaveraceae species need to be sequenced to analyze their variations and evolution.

Approximately 10% of the plant mt genomes that have been sequenced contain multiple chromosome structures (Wu et al., 2022). Recombination events are known to alter the abundance of sub-genomic molecules (Preuten et al., 2010). In the draft map of the *C. saxicola* mt genome, the coverage of contig 3 was nearly double that of either contig 1 or contig 2 (**Supplementary Figure S1**; **Supplementary Table S2**), indicating that the recombination configurations such as two smaller circles might be present in the *C. saxicola* mitochondria. In contrast to animal mt genomes, which exhibit very few rearrangements, continuous rearrangements are known to occur in plant mt genomes (Gualberto and Newton, 2017). These may be caused directly by continuous fusion and fission of plant mitochondria, resulting in the reunification of both damaged and undamaged mtDNA (Lannera and Connor, 2001; Gualberto and Newton, 2017). This would significantly decrease the number of point mutations, and potentially increase the longevity of certain plants (Lannera and Connor, 2001; Gualberto and Newton, 2017). Frequent intermolecular rearrangements of mtDNA might also have occurred in *C. saxicola* mitochondria, as evidenced by the number of SSRs, TR and dispersed repeats identified in the *C. saxicola* mt genome (**Supplementary Figure S3**; **Supplementary Tables S6**-**S8**). To date, whether these repetitive elements redundant or functional are largely unknown, but they determine the mt genome size to some extent (Putintseva et al., 2020; Wu et al., 2022). Additionally, whether and when multiple conformations exist and what functions they carry out also require further investigations.

In plants, mt genomes not only rearrange with each other, but also communicate with plastids, nuclei and even other species (Mower et al., 2012). Here, we identified a total of 12 homologous fragments between the mt and ct genomes of *C. saxicola* (**Figure 3**), accounting for 1.04% of the entire mt genome (**Supplementary Table S9**). These fragments contained PCGs, tRNA genes and intergenic regions of the plastid genome (**Supplementary Table S9**). Compared to many other species, the proportion of MTPTs was relatively low between the mt and cp genomes of *C. saxicola* (Jiang et al., 2023a; Jiang et al., 2023b; Shan et al., 2023). It concluded that plastid-related sequences are not responsible for the mt genome size (Putintseva et al., 2020), however, MTPTs and nuclear mitochondrial transferred fragments are crucial for mt genome evolution (Wang D. et al., 2012; Jiang et al., 2023b). Interestingly, all the tRNA genes contained in MTPT fragments were complete (Jiang et al., 2023a; Jiang et al., 2023b; Shan et al., 2023), indicating that they may still be functional. However, some of the transferred PCGs were incomplete (**Supplementary Table S9**), and this phenomenon has also been observed in other species (Jiang et al., 2023a). Whether these PCGs are still functional is an open question.

### 4.2 RNA editing events in the mt *C. Saxicola* genome

RNA editing, resulting in the post-translational insertion, deletion and conversion of nucleotides into coding and non-coding regions of RNA molecules, is known to occur in the nucleus, cytoplasm and DNA-containing organelles (Covello and Gray, 1989; Hiesel et al., 1989; Maier et al., 1996; Maldonado et al., 2022). Since its discovery in 1986 (Benne et al., 1986), RNA editing has been identified in animals, plants, lycophytes, ferns, protozoa and some viruses, with most organisms exhibiting variable preferred types (Hao et al., 2021). In particular, the mitochondria and chloroplasts of land plants exhibit extensive RNA editing, with C/U editing being the most common (Small et al., 2020). Furthermore, mitochondria contain approximately 20 times more RNA editing sites than plastids (Giegé and Brennicke, 1999). In both mitochondria and chloroplasts, RNA editing is involved in male sterility, seed development, environmental adaptation and pathogen resistance (García-Andrade et al., 2013; Yan et al., 2018; Yang et al., 2020). Although several methods have been developed for the detection of RNA editing sites and levels, Sanger sequencing is still the most reliable.

Here, a total of 779 ‘C to U’ RNA editing sites were predicted in the 40 C*. saxicola* mt PCGs by Deepred-mt (**Figure 7**; **Supplementary Table S12**) (Edera et al., 2021). Among these predicted RNA editing sites, 506 (65.0%) were validated using a combination of PCR amplification and Sanger sequencing (**Supplementary Figure S4**). Additionally, 38 other RNA editing sites were validated but not predicted (**Supplementary Table S12**). Several reasons may be responsible for these discrepancies: (1) the deep RNA sequencing data of 21 plant mt genomes, selected for the training dataset of Deepred-mt, might be not as comprehensive as possible to predict all ‘C to U’ RNA editing sites in variable plant mt genomes; (2) although Deepred-mt exhibits higher precision in the prediction of RNA editing sites compared to other two state-of-the-art predictive methods, PREP-Mt (Mower, 2009) and PREPACT (Lenz et al., 2018), the accuracy is not 100%, and the deviation might be due to efficient but still imperfect learning strategies or other factors used in computer simulation (Edera et al., 2021). In all, 546 ‘C to U’ RNA editing sites were validated, 25 (4.6%) of which were synonymous substitution sites (**Supplementary Table S12**). This proportion is similar to the mt genome of *Taraxacum mongolicum*, in which validated synonymous conversions accounted for 4.69% (10/216) (Jiang et al., 2023a). Among the 25 synonymous substitution sites, 21 occurred on the third bases of triplet codons, and the rest occurred on the first bases of triplet codons. These four synonymous conversions were identified in *atp9* (base position 209), *mttB* (base position 37), *nad1* (base position 823) and *rps10* (base position 7), and all represented ‘Leu to Leu’ substitutions (**Supplementary Table S12**). The majority of RNA editing sites occurred in the first and second positions of triplet codons, resulting in synonymous substitutions (**Supplementary Table S12**). This phenomenon has also been observed in *A. thaliana* (Giegé and Brennicke, 1999). Among the mt PCGs, *nad4* contained the greatest number of RNA editing sites (54), while *mttB* and *ccmB* contained 46 and 43 editing sites, respectively (**Supplementary Tables S12**, **S13**). Nad4 and the other eight mt nad proteins (nad1, nad2, nad3, nad4L, nad5, nad6, nad7 and nad9) are components of complex I, which are the largest of the respiratory complexes found in the mitochondria of nearly all land plants (Møller et al., 2021). Multiple RNA editing sites were validated among the *nad* genes (**Supplementary Tables S12**, **S13**), indicating that post-transcriptional modifications like RNA editing may be crucial to the functioning of complex I. Interestingly, analysis of the RNA-editing sites from 17 plant species also showed that *nad4* contained the greatest number, consistent with our results (**Figure 7**, **Supplementary Tables S12**, **S13**) (Maldonado et al., 2022). Notably, the number of RNA editing sites appears to be disproportionate to the length of the gene (**Supplementary Table S11**) (Maldonado et al., 2022; Jiang et al., 2023a; Li et al., 2023).

Furthermore, two stop codons and one traditional start codon (AUG) were found to be introduced by RNA editing events (**Supplementary Table S12**). One of the stop codons occurred in *atp9* and another occurred in *rps10*. Both events were CGA to UGA, resulting in the conversation of Arg into a stop codon (**Supplementary Table S12**). The validated conversion of ACG to AUG introduced a traditional start codon into *nad4L* (**Supplementary Table S12**). It should be noted that unedited ACG may also serve as a start codon in plant plastids and mitochondria (Dong et al., 1998; Zandueta-Criado and Bock, 2004). Studies have suggested that C-to-U editing results in a reduced rate of TT-dimers in DNA, increasing the UV resistance of land plants (Binder and Brennicke, 2003; Fujii and Small, 2011). Whether the conversion of ACG to AUG influences translation efficiency requires further study.

### 4.3 Transcript profiles of 24 core *C. saxicola* mt PCGs

Most mitochondrial genes are combined with other unrelated genes into poly-cistronic primary transcripts, and endo- and exo-nucleolytic reactions process the transcripts (Møller et al., 2021). RT-qPCR indicated that the expression of 24 core *C. saxicola* mt PCGs differed among the different organs (**Figure 8**). Notably, the majority exhibit relatively higher expression in aerial tissues, including stems, branches, and mature and young leaves, but minimal expression in the roots and flowers (**Figure 8**). Interestingly, cavidines, especially dehydrocavidine, are known to be present mostly in the roots of *Corydalis* species (Xu et al., 2022b). Our studies suggest that the content of dehydrocavidine is highest in flowers, followed by the roots (data not shown). Whether these results represent correlation or coincidence requires further investigation.

In plants, Ca^2+^ not only provides structural stability, but also acts as a dynamic intracellular messenger involved in stress response and development (He et al., 2021; Pirayesh et al., 2021). Ca^2+^ was also demonstrated to be involved in the biosynthesis of secondary metabolites (Martins et al., 2021; Ahmad et al., 2022). As a plant native to karst regions, *C. saxicola* is particularly resistant to calcium. In addition, our work indicates that high Ca^2+^ concentrations result in a significant increase in the dehydrocaverdine content of the roots of *C. saxicola* (**Supplementary Figure S5**). Research has revealed a physiological relationship between Ca^2+^ and mitochondrial function (Pirayesh et al., 2021). To investigate whether and to what extent the *C. saxicola* mt PCGs are responsive to exogenous CaCl_2_ treatment, we performed RT-qPCR to analyze the relative expression of 24 core mt PCGs in *C. saxicola* roots. Interestingly, the majority of these genes exhibited upregulated expression in response to exogenous CaCl_2_ treatment (**Figure 9**). These results demonstrate that high concentrations of exogenous Ca^2+^ influence the mitochondrial function in *C. saxicola*, as all 24 core mt genes were components of the mitochondrial electron transport chain (Møller et al., 2021). It has been reported that the concentration of [Ca^2+^] _mito_ is approximately double that of [Ca^2+^]_cyt_ under stress, and that entry of these cations into the mitochondria is critical to prevent its accumulation in the cytosol (He et al., 2021; Pirayesh et al., 2021). However, whether the concentration of [Ca^2+^] _mito_ in *C. saxicola* roots is higher than that of [Ca^2+^]_cyt_ still needs to be investigated. Furthermore, the expression of nuclear-encoded components should be analyzed to validate the functional elevation of respiratory complexes. Additionally, whether the upregulated expression of these mt PCGs in response to exogenous CaCl_2_ resulted in increased dehydrocaverdin biosynthesis requires further study. One possible explanation is that O-methyltransferases, the key enzymes involved in the biosynthesis of dehydrocaverdin, require ATP to transfer the methyl group from S-adenosylmethionine to its specific substrates (Xu et al., 2022b). Recently, highly efficient mt genome-editing techniques have been developed (Kang et al., 2021; Nakazato et al., 2022; Forner et al., 2023), which will facilitate the functional validation of mt genes.

## 5 Conclusions

In this study, we mapped out the mt genome of *C. saxicola*, an endangered medicinal plant endemic to karst habitats. Variable aspects of the mt genome have been investigated, including the basic composition, codon preference, repeat sequences, MTPTs and genome comparisons with those of closely related species. These discoveries can be employed to create molecular markers and assist scientists in comprehending the evolution and how to classify the plants within the *Corydalis* species.

We performed an extensive analysis of the RNA editing events in 40 C*. saxicola* mt PCGs and validated 70% (65% predicted ones and 5% were unpredicted) of these by using PCR amplification and Sanger sequencing. Additionally, we transcriptionally profiled 24 core mt PCGs in different tissues of *C. saxicola*. The results showed the majority were minimally expressed in roots and flowers. Interestingly, most of these genes exhibited upregulated expression in response to exogenous CaCl_2_ treatment. These investigations will be helpful in understanding the biological functions of the mt PCGs towards variable biotic and abiotic stresses.

## Data availability statement

The datasets presented in this study can be found in online repositories. The names of the repository/repositories and accession number(s) can be found in the article/**Supplementary Material**.

## Ethics statement

Fresh and healthy *C. saxicola* tissues were collected for this study. *C. saxicola* was identified by Z-JZ and CL of GBGMP (Nanning, Guangxi Zhuang Autonomous Region, China). Voucher specimens (reference number: 450123130505040LY) were deposited in the GBGMP Herbarium. This study, including plant sample collection, complies with relevant institutional, national and international guidelines and legislation. No specific permits were required for plant collection.

## Author contributions

CL: Writing – original draft, Data curation, Formal Analysis, Funding acquisition, Investigation, Methodology, Resources, Software. HL: Data curation, Formal Analysis, Investigation, Methodology, Software, Writing – original draft. MQ: Validation, Writing – review & editing. Y-JT: Validation, Writing – review & editing. X-LO: Validation, Writing – review & editing. X-YC: Formal Analysis, Writing – original draft. YW: Formal Analysis, Resources, Writing – review & editing. Z-JZ: Conceptualization, Investigation, Writing – review & editing. ML: Conceptualization, Data curation, Investigation, Methodology, Software, Writing – original draft, Writing – review & editing.

## Supplementary material

Supplementary Figure 1 The uniting graph of the mt genome of *C. saxicola* obtained from the *de novo* assembly of Illumina reads. Three contigs (ctg1, ctg2 and ctg3) were found and these are depicted in different colors in the graph.

Supplementary Figure 2 The cp genome map of *C. saxicola*. Genes drawn inside and outside the circle are transcribed clockwise and counterclockwise, respectively. Genes belonging to different functional groups are color-coded. The light and darker gray in the inner circle correspond to Adenine-Thymine (AT) and GC content, respectively. LSC, large single-copy; SSC, small single-copy; IR, inverted repeat.

Supplementary Figure 3 Repeated sequences in the *C. saxicola* mt genome. **(A)** Distribution of SSRs in the *C. saxicola* mt genome. **(B)** Distribution of tandem and dispersed repeats in the *C. saxicola* mt genome.

Supplementary Figure 4 Agarose gel electrophoresis of gDNA and cDNA PCR products of 40 C*. saxicola* mt PCGs. The first well was loaded with 5 µL of marker, and the rest of the wells were loaded with 10 µL of PCR products for each gene. M: marker; I, II, III, IV, and V: exon-contained gene fragments.

Supplementary Figure 5 The content of dehydrocavidine in variable organs of *C. saxicola* treated with different concentrations of CaCl_2_. Different lowercase letters indicate significant differences among treatments (ANOVA); differences were considered significant at *p* < 0.05.
