# Correction: RNA editing events and expression profiles of mitochondrial protein-coding genes in the endemic and endangered medicinal plant, *Corydalis saxicola*

**DOI:** 10.3389/fpls.2026.1783725

**Published:** 2026-01-23

**Authors:** Cui Li, Han Liu, Mei Qin, Yao-jing Tan, Xia-lian Ou, Xiao-ying Chen, Ying Wei, Zhan-jiang Zhang, Ming Lei

**Affiliations:** 1 National Center for Traditional Chinese Medicine (TCM) Inheritance and Innovation, Guangxi Botanical Garden of Medicinal Plants, Nanning, China; 2 Guangxi Key Laboratory of Medicinal Resources Protection and Genetic Improvement, Guangxi Botanical Garden of Medicinal Plants, Nanning, China; 3 Guangxi Engineering Research Center of Traditional Chinese Medicine (TCM) Resource Intelligent Creation, Guangxi Botanical Garden of Medicinal Plants, Nanning, China; 4 School of Basic Medical Sciences, Guangxi Medical University, Nanning, China; 5 Guangxi Key Laboratory for High-Quality Formation and Utilization of Dao-di Herbs, Guangxi Botanical Garden of Medicinal Plants, Nanning, China

**Keywords:** *Corydalis saxicola*, mitochondrial genome, RNA editing, mitochondrial plastid DNAs, expression profile

There was a mistake in the Funding statement, an incorrect number was used. The correct statement is:

Funding

The author(s) declare financial support was received for the research, authorship, and/or publication of this article. This research was funded by the Natural Science Foundation of Guangxi (2023GXNSFAA026509); the Guangxi Science and Technology Base and Talent Project (Guike AD22080016); Guangxi Key R&D Program (Guike AB23026092); Guangxi Key Laboratory of Medicinal Resources Protection and Genetic Improvement (KL2022ZZ04, KL2023ZZ09); Guangxi Appropriate Technology Development and Promotion Project of Traditional Chinese Medicine (GZSY22-02) and Key Techniques Research and Promotion of Guangxi Medicinal Materials Varieties (GZKJ2314). The funders were not involved in the study design, data collection, analysis, publication decision and manuscript preparation.

