# Supplementary figures and images for: RNA editing events and expression profiles of mitochondrial protein-coding genes in the endemic and endangered medicinal plant, Corydalis saxicola
Source: Front Plant Sci. 2024 Feb 6;15:1332460. doi: 10.3389/fpls.2024.1332460 (PMC10876856; doi:10.3389/fpls.2024.1332460)

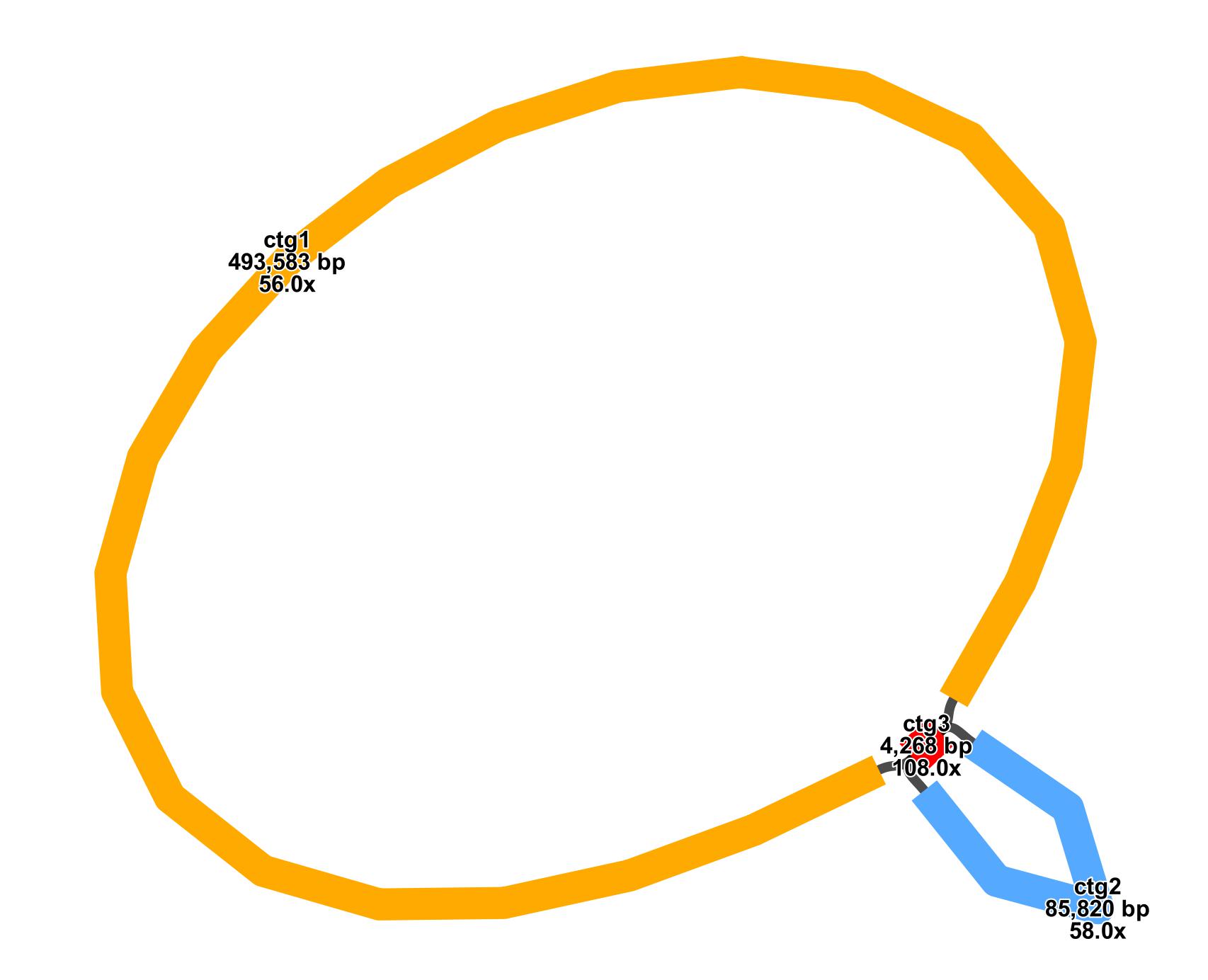

Supplement: Supplementary Figure 1 — The uniting graph of the mt genome of C. saxicola obtained from the de novo assembly of Illumina reads. Three contigs (ctg1, ctg2 and ctg3) were found and these are depicted in different colors in the graph. [file Image_1.jpeg]

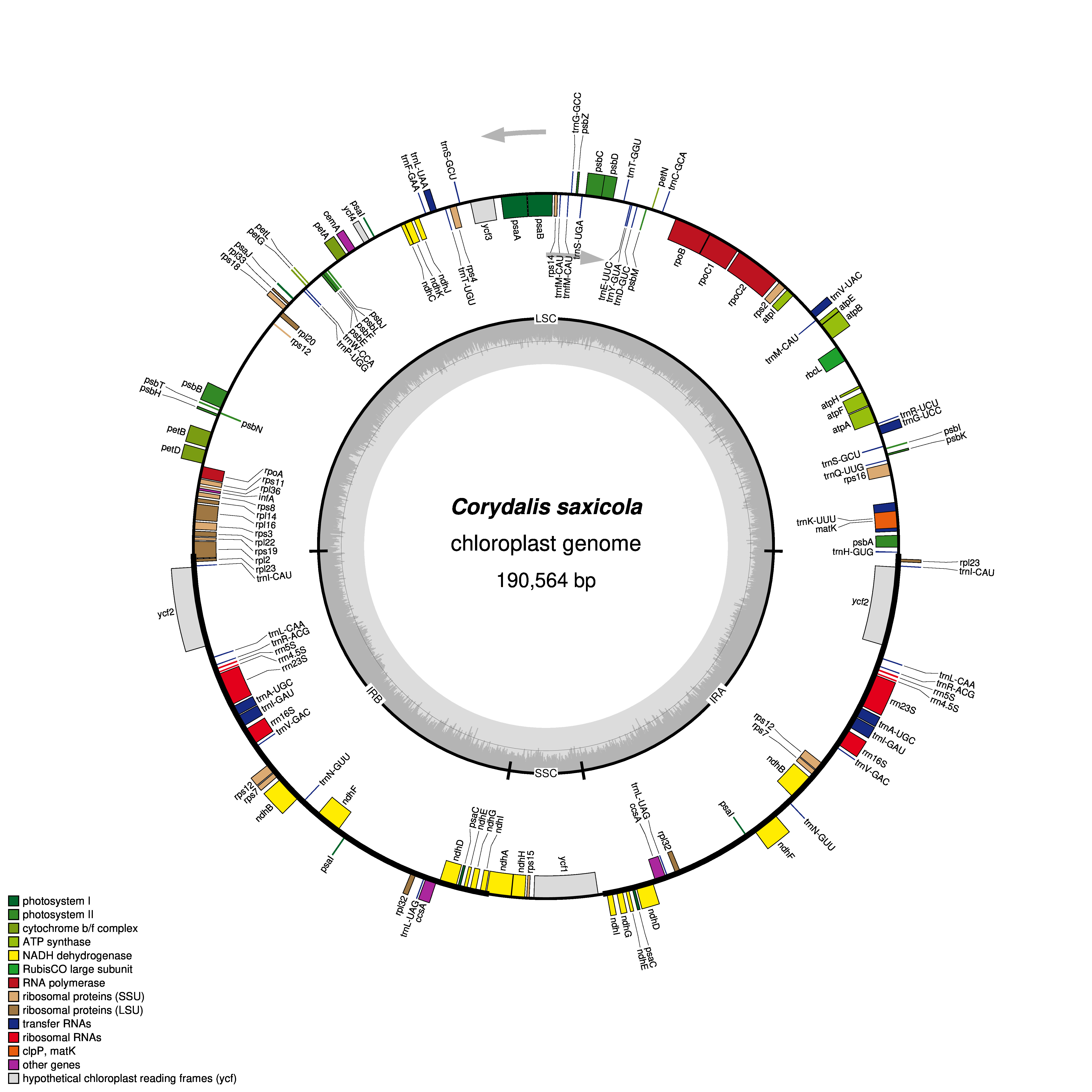

Supplement: Supplementary Figure 2 — The cp genome map of C. saxicola. Genes drawn inside and outside the circle are transcribed clockwise and counterclockwise, respectively. Genes belonging to different functional groups are color-coded. The light and darker gray in the inner circle correspond to Adenine-Thymine (AT) and GC content, respectively. LSC, large single-copy; SSC, small single-copy; IR, inverted repeat. [file Image_2.jpeg]

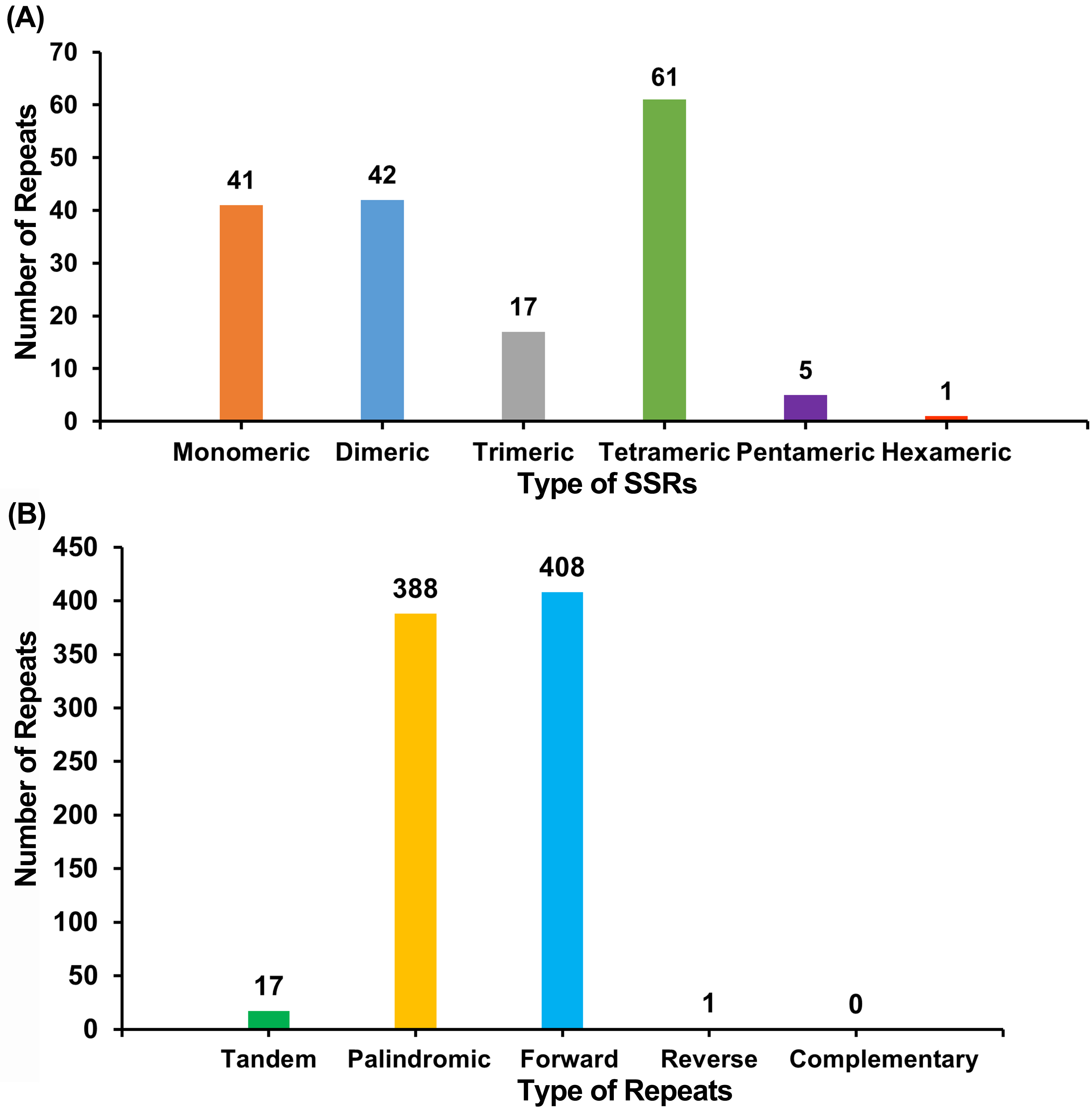

Supplement: Supplementary Figure 3 — Repeated sequences in the C. saxicola mt genome. (A) Distribution of SSRs in the C. saxicola mt genome. (B) Distribution of tandem and dispersed repeats in the C. saxicola mt genome. [file Image_3.jpeg]

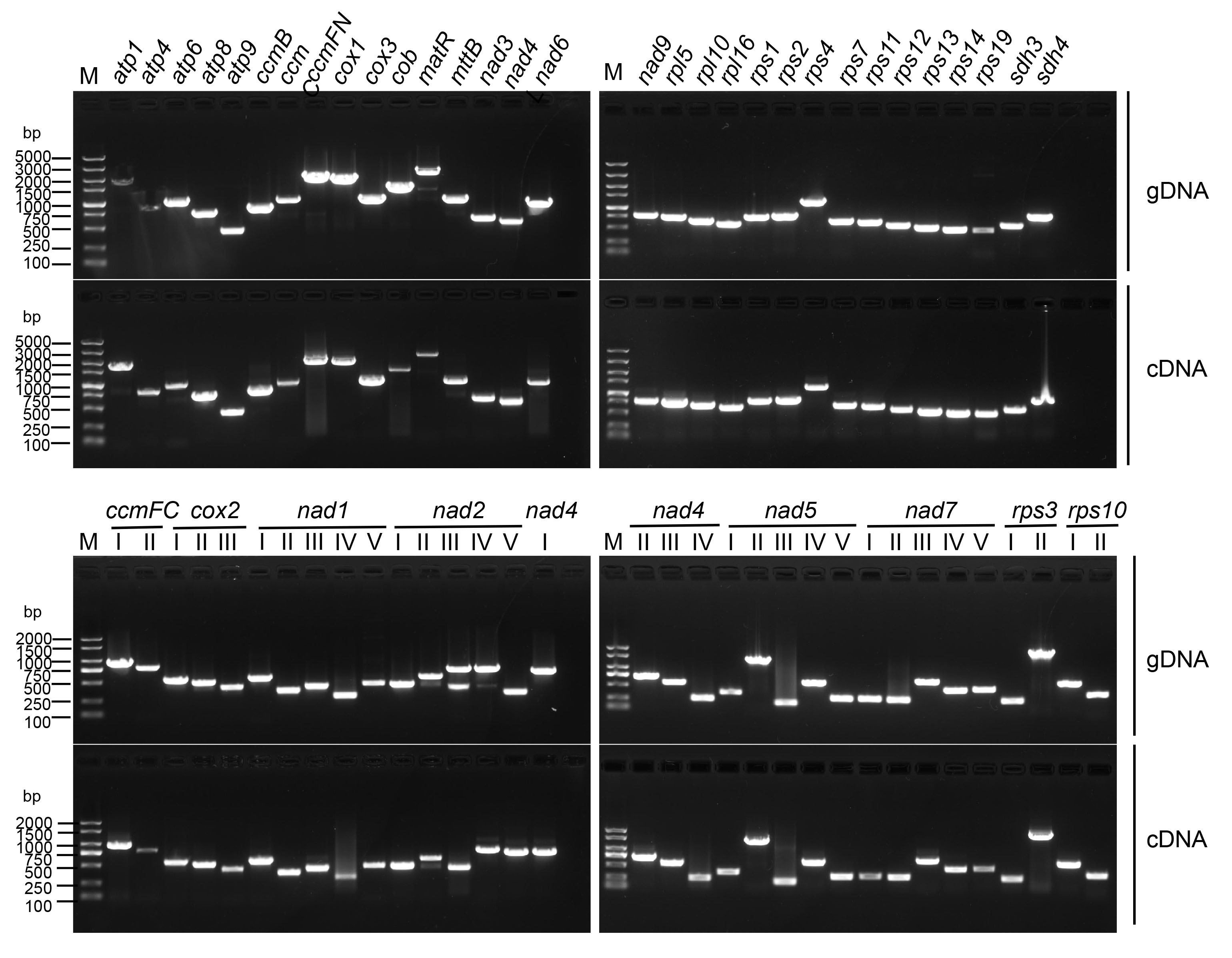

Supplement: Supplementary Figure 4 — Agarose gel electrophoresis of gDNA and cDNA PCR products of 40 C. saxicola mt PCGs. The first well was loaded with 5 µL of marker, and the rest of the wells were loaded with 10 µL of PCR products for each gene. M: marker; I, II, III, IV, and V: exon-contained gene fragments. [file Image_4.jpeg]

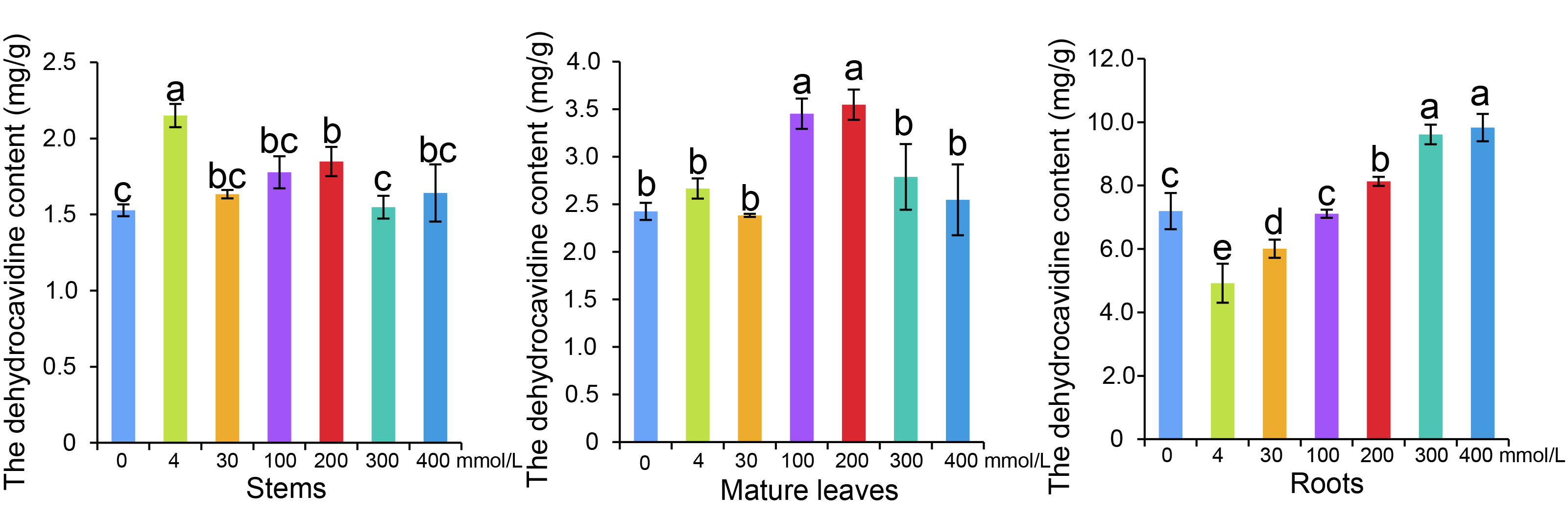

Supplement: Supplementary Figure 5 — The content of dehydrocavidine in variable organs of C. saxicola treated with different concentrations of CaCl2. Different lowercase letters indicate significant differences among treatments (ANOVA); differences were considered significant at p < 0.05. [file Image_5.jpeg]
